# Discrepancy between subjective and objective memory change after epilepsy surgery: Relation with seizure outcome and depressive symptoms

**DOI:** 10.3389/fneur.2022.855664

**Published:** 2022-07-22

**Authors:** Florian Johannes Mücke, Marc Petrus Hendriks, Christian Günther Bien, Philip Grewe

**Affiliations:** ^1^Department of Epileptology (Krankenhaus Mara), Bielefeld University, Bielefeld, Germany; ^2^Department of Neuropsychology and Rehabilitation Psychology, Donders Institute for Brain, Cognition and Behavior, Radboud University Nijmegen, Nijmegen, Netherlands; ^3^Academic Centre of Epileptoloy, Kempenhaeghe, Heeze, Netherlands; ^4^Clinical Neuropsychology and Epilepsy Research, Medical School EWL, Bielefeld University, Bielefeld, Germany

**Keywords:** epilepsy surgery, neuropsychology, subjective memory, verbal memory, quality of life, seizures, depressive symptoms

## Abstract

Complaints pertaining to memory functioning are among the most often reported cognitive symptoms in patients with epilepsy. However, research suggests a considerable mismatch between patients' perception of memory functioning and the objective performance as measured with standardized neuropsychological tests. Depressive mood might be an important factor in explaining this discrepancy, though other variables have also occasionally been reported as relevant. There are mixed results as to which role these factors play in determining the overall quality of life of patients with epilepsy. The present study aimed to quantify the mismatch between subjective and objective memory functioning by taking into account the dynamic change of these factors as well as depressive symptoms after epilepsy surgery. Moreover, the influencing factors of subjective and objective memory change were investigated as well as their effects on the overall quality of life. Pre- and postoperative data from 78 patients with focal epilepsy were retrospectively analyzed. The results showed that (1) patients with clinically relevant postoperative depressive symptoms underestimate their actual memory performance; (2) for non-seizure-free patients, a postoperative decrease in depressive symptoms was associated with a tendency to underestimate memory decline; (3) the relationship between objective memory change and quality of life is mediated by the factors subjective memory change and depressive mood. Our data demonstrate a quantitative approximation of a pronounced depression-related negative biased self-perception of memory functioning of roughly 1 to 1.5 standard deviations. Moreover, it seems that when patients are relieved of having recurrent epileptic seizures, they may be less influenced by depressive symptoms when judging their memory change. Taken together, our study demonstrates the clinical relevance of incorporating subjective measures of memory functioning and mood that go beyond objective memory performance for the interpretation of how changes in memory functioning may affect patients' quality of life after epilepsy surgery.

## Introduction

In the last decades, neuropsychological research has given us extensive insights into memory functioning in patients with epilepsy (PWE) after epilepsy surgery (1). Effort has also been spent to clarify the subjective perception of memory complaints in PWE. When comparing objective and subjective memory, PWE often tend to misjudge their performances (2–9). It has been suggested that depressed mood (10–13) and psychological distress (4) are factors that might explain this discrepancy, both reflecting an inability to cope with or adjust to the condition (5).

Only a few studies considered these factors and tried to establish an integrative approach to subjective and objective memory functioning as well as psychosocial well-being in PWE over the course of the condition, especially prior to and after epilepsy surgery. In their review, Sherman and colleagues reported on three studies that measured self-reported subjective changes in cognition after epilepsy surgery. Subjective memory loss was evident in 8–20%, whereas memory gains were described in 11–52% of patients. Interestingly, objective memory decline was found in 20–44% of patients (1) suggesting a discrepancy between subjective and objective measures. In line with this, other surgical outcome studies in epilepsy cohorts only found small or no associations at all between subjective and objective memory measures and consistently suggest levels of depression as an indicator for postoperative subjective memory decline (7, 14–17). It has been argued that postoperative mood and subjective memory complaints may also be related to seizure status (14, 18) or medication side effects (14, 15). It may be reasoned that, from a clinical standpoint, subjective memory impacts the psychosocial well-being of PWE more strongly than actual cognitive functioning. Therefore, subjective memory complaints are often disregarded as an ‘add-on' in neuropsychological assessment and are often considered as having an inconclusive value in the diagnostic process, because of the many potential factors they interact with.

We argue, however, that there is a substantial need to consider subjective memory complaints pre- and postoperatively since, firstly, it may have decisive value for the individual patient and his or her overall quality of life (QoL). Secondly, it provides the clinician with important additional information not only about emotional well-being but also about its interaction with the cognitive status prior to and after surgery. Thus, for counseling patients for epilepsy surgery and for further planning of psychological treatment options, it seems imperative to disentangle the relationship between subjective and objective memory performance as a function of mood and the implications of it for the overall QoL.

The present study investigated (1) the relationship between mood and pre- to postoperative subjective and objective memory functioning in PWE; (2) how the factor seizure status relates to the change of subjective and objective memory functioning and postoperative mood; (3) the relationship between objective memory change and the overall QoL. We hypothesized that (1) depressed patients do more often report subjective memory deficits (15, 17) and may also overestimate their memory decline from pre- to postoperative assessment. In contrast, there should be no major difference in objective memory performance between depressed and non-depressed patients (5, 19). We further expected that (2) seizure freedom may moderate the association between subjective memory, objective memory, and depressive symptoms (18); patients with ongoing seizures who experience a decrease in depressive symptoms might tend to underestimate their memory decline. Finally, we hypothesized that (3), QoL will not only be affected by depressive mood but by an interplay of this variable with subjective and objective memory scores (5, 20).

## Materials and methods

### Participants

The study included 78 patients (35 female/43 male) with focal epilepsy who underwent extensive interictal and ictal preoperative video-electroencephalography (EEG) monitoring and epilepsy surgery at the Epilepsy Center Bethel in Bielefeld, Germany (21, 22). Inclusion criteria were: (1) diagnosis of focal epilepsy confirmed by EEG, seizure semiology, and MRI findings during presurgical diagnostics (2), availability of results from standard pre- and postoperative neuropsychological assessment (including self-ratings of cognitive functioning and depressive symptoms), (3) availability of self-ratings of overall QoL at the 24-month follow-up between February 2016 and March 2018, (4) age at examination of at least 18 years.

Resection side and type of surgical procedure were specified based on neuroradiological findings and scalp or invasive video-EEG recordings. Seizure freedom was defined as sustained seizure freedom, with or without focal aware seizures, 24 months after surgery (Engel class 1A and 1B) (23). For demographic and clinical characteristics, see Table 1. The study was carried out in compliance with the Code of Ethics of the World Medical Association (Declaration of Helsinki). All patients gave written informed consent to participate in this study. The study protocol was approved by the local ethics committee (University of Bielefeld, Germany, no. 2016-001).

**Table 1:** Medical and demographic characteristics of the patient sample.

**Variables**		***N*** = **78**
Sex (female)	*n* (%)	35 (44.9)
Age at surgery (years)	M (SD)	39.4 (13.9)
Age at epilepsy onset (years)	M (SD)	16.9 (14.0)
Duration of epilepsy (years until surgery)	M (SD)	22.3 (15.2)
Side of surgery (left)	*n* (%)	28 (35.9)
Site of surgery (non-TLE)	*n* (%)	22 (28.2)
Seizure outcome (Engel 1A/1B)^*a*^	*n* (%)	47 (61.8)
ASM polytherapy (>1 drug)	*n* (%)	
preoperative		64 (82.1)
postoperative		41 (53.2)
ADM therapy (≥1 drug)	*n* (%)	
preoperative		12 (15.4)
postoperative		14 (18.2)

*TLE, temporal lobe epilepsy; M, mean; SD, standard deviation; ASM, anti-seizure medication; ADM, anti-depressive medication*.

^a^*for three patients, outcome data were carried forward from the 6-months-follow-up assessment; for one patient, outcome was based on a 3.5-year-follow-up assessment*.

### Measures

#### Objective memory measures

From our standard pre- and postsurgical neuropsychological test battery, we examined test results from the Verbal Learning and Memory Test (VLMT) (24), which is the German adaption of the Rey Auditory Verbal Learning and Memory Test (25). The procedure of this test has previously been described elsewhere (26). Parameters of interest in our study were learning capacity (sum of correctly remembered words throughout trial 1 to trial 5), long-term recall (number of correctly recalled words after a 30-min delay), long-term retention (number of correctly remembered words after five learning trials minus the number of correctly remembered words after a 30-min delay), and long-term recognition (number of correctly recognized words after a 30-min delay). Scores on each variable were transformed into standardized z-scores according to the normative scores of the VLMT. We then calculated the mean of those four z-transformed variables to obtain an overall z-score indicating the overall verbal memory capacity.

#### Subjective cognitive measures

For the assessment of subjective cognitive functions, patients had to fill in the “Fragebogen zur geistigen Leistungsfähigkeit” (FLEI; Questionnaire for complaints of cognitive disturbances) (27). The FLEI consists of 30 items that assess everyday situations with demands on cognitive functions. Items are divided into three subscales, (i.e., attention, memory, and executive functions). For the purpose of our study, we only included data from the memory subscale (10 items; Cronbach's α = 0.92) in our analyses. The participant has to rate each situation in terms of the frequency of experienced disturbances in each of the situations during the past 6 months. Answers are given on a five-point scale (i.e., “never,” “rarely,” “occasionally,” “frequently,” and “very frequently”). For an *ad-hoc* translated English version of the FLEI, the reader is referred to (4). The FLEI has been shown to reliably detect subjective cognitive complaints in patients with schizophrenia and depression relative to healthy controls (all three subscales: Cronbach's α ≥ 0.91, r_split−half_ ≥ 0.87) (27). As with the objective measures, scores were z-transformed to obtain comparability.

#### Depressive symptoms

For the assessment of depressive symptoms, patients were asked to fill in the BDI-II (28). The German version of the BDI-II consists of 21 items (Cronbach's α = 0.93; r_test−retest_ = 0.78) assessing the severity of depressive symptoms (29). It has been thoroughly validated in various patient samples (30). In our study, patients with a BDI-II score below 14 were classified as clinically non-depressed, whereas patients with scores of 14 or higher were classified as having clinically relevant depressive symptoms (29).

#### Quality of life

For the evaluation of the QoL, patients were asked to fill in the German version of the “Quality of life in epilepsy” questionnaire (QOLIE-31) (31, 32). The QOLIE-31 includes 31 items assessing QoL in PWE. It is an often-used instrument that has been validated in various patient populations and translated into many different languages (33–35). The overall total score-subscale of the QOLIE-31 includes several items on cognition and emotional well-being. As one might expect high intercorrelations between these items and the self-rating instruments of our study (i.e., BDI-II, FLEI), we did not analyze the overall total score-subscale to prevent redundancy caused by these intercorrelations. Rather, we aimed to detect the specific contributions of objective memory, subjective memory, depression, and QoL, by only analyzing data from the overall QoL scale, which comprises two items asking patients only about their overall QoL (α = 0.79; r_test−retest_ = 0.84).

### Data analysis

To analyze pre- to postoperative change in subjective vs. objective memory in dependance of depressive symptoms, we performed repeated-measures analyses of variance (ANOVA) using depressive symptoms (“depressed” vs. “non-depressed”) and measure (objective vs. subjective) as between-subject factors and time (pre vs. post) as within-subject factor. The mean z-score of all VLMT variables was computed to serve as the dependent variable.

To determine the role of seizure frequency in the pre- to postoperative change of subjective and objective memory as well as depressive symptoms, we first computed change scores (i.e., z-score_post_ minus z-score_pre_) of the factors subjective and objective memory as well as depressive symptoms. A statistically significant change was based on reliable change indices with 90% confidence intervals. We then calculated the difference between the variables subjective and objective memory change (i.e., subjective minus objective) to get an indicator for the discrepancy between subjective and objective scores. This difference served as a measure of the extent of over- or underestimation of memory change in relation to objective memory change; negative values represent overestimation, whereas positive values represent an underestimation of memory decline, respectively; values near zero represent an adequate estimation of memory change. Subsequently, we calculated Pearson product-moment correlation coefficients between the discrepancy of subjective vs. objective memory change on the one hand, and change of depressive symptoms, on the other hand separately for seizure-free and non-seizure-free patients.

For our third research question, we performed a serial mediation analysis (36, 37) to model the relationship between memory decline and QoL and the mediating influence of the two variables subjective memory change and depressive symptoms. Memory decline was calculated as a dichotomous variable (i.e., decline vs. no decline); a decline of memory functions was defined for change scores ≤ 3 (i.e., raw score_post_ minus raw score_pre_ ≤ 3) for the long-term recall of the VLMT corresponding to a statistically significant change based on the 90%-reliable change index (24). Both mediator variables, namely subjective memory change and depressive symptoms represented z-scores. QoL-scores represented raw scores of the overall QoL scale from the QOLIE-31.

Statistical analyses were conducted using SPSS Statistics 25 (IBM, Chicago, USA). The serial multiple mediation analysis was conducted using the PROCESS macro for SPSS, Version 5.5.3 (36, 37). Figures were produced using Matlab R2020a (The Mathworks, Natick, USA) and Microsoft Excel (Microsoft Corporation, Redmond, USA).

## Results

In the following sections, we report the results of the above-mentioned analyses with respect to the three research questions outlined in the introduction of this paper. For an overview of means and standard deviations of all outcome variables at pre- and postoperative assessment, the reader is referred to Table 2.

**Table 2:** Pre- and postoperative mean outcomes and mean differences in subjective and objective memory performance as well as severity of depressive symptoms and quality of life.

**Parameter**	**Pre**			**Post**			**Diff**.		
	**Total**	**Depr**	**Non-depr**	**Total**	**Depr**	**Non-depr**	**Total**	**Depr**	**Non-depr**
Subjective memory (FLEI)	−0.47^63^ (0.15)	−1.29^13^ (0.33)	−0.26^50^ (0.16)	−0.27 (0.20)	−1.99 (0.50)	0.18 (0.18)	0.21 (0.19)	−0.70 (0.39)	0.44 (0.21)
Objective memory (VLMT)	−0.23^74^ (0.10)	−0.22^15^ (0.29)	−0.24^59^ (0.10)	−0.05 (0.11)	−0.30 (.28)	0.01 (0.12)	0.18 (0.11)	−0.08 (0.23)	0.24 (0.12)
Depressive Symptoms (BDI-II)	−0.45^60^ (0.15)	−1.14^12^ (0.35)	0.28^48^ (0.15)	−0.06 (0.14)	−1.87 (0.15)	0.40 (0.09)	0.39 (0.15)	−0.73 (0.32)	0.67 (0.15)
Quality of life (QOLIE-31)	n.a.	n.a.	n.a.	72.26^73^ (20.27)	54.64^14^ (28.27)	76.44^59^ (15.43)	n.a.	n.a.	n.a.

*Means and standard deviations are given in standardized z-scores, except for the QOLIE that is presented in raw scores. Standard deviations appear in parentheses. Superscript numbers represent the observed n of the respective variable and group (applies to pre- as well as post-operative data). FLEI, Fragebogen zur geistigen Leistungsfähigkeit (Questionnaire for complaints of cognitive disturbances); VLMT, Verbal Learning and Memory Test; BDI-II, Beck Depression Inventory; QOLIE, Quality of life in epilepsy questionnaire; Pre, preoperative assessment; Post, postoperative assessment; Diff., difference (Post – Pre); depr, depressed*.

### Memory and depressive symptoms

**Figure 1:**
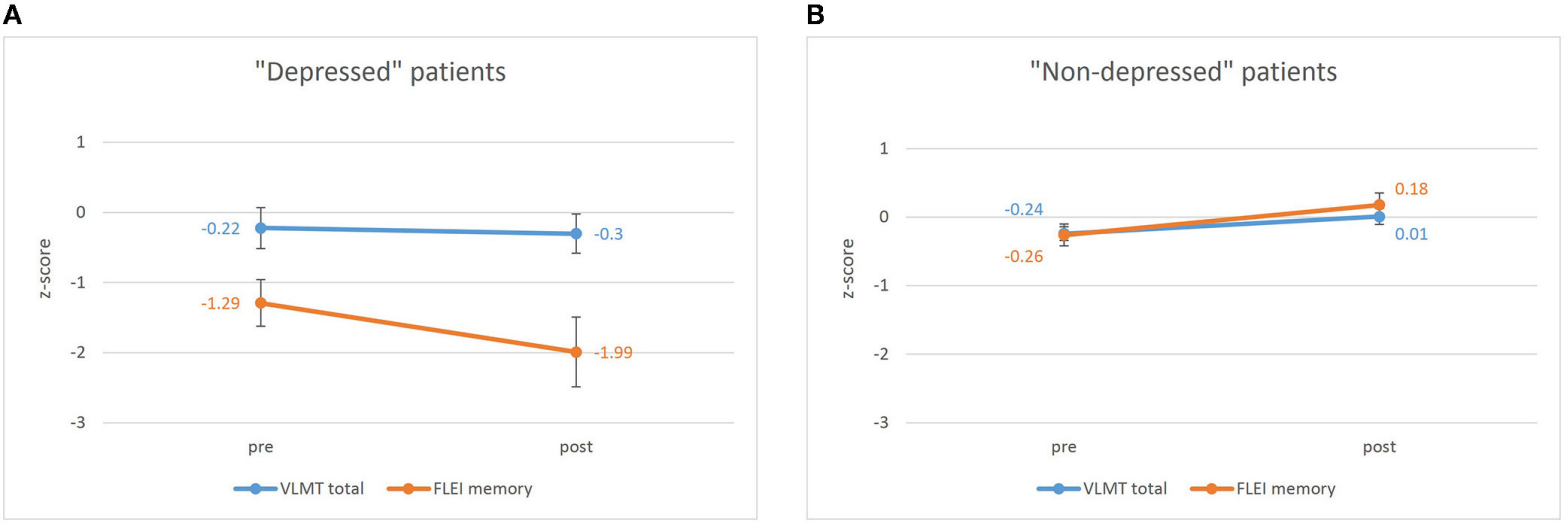
Pre- to postoperative results of the VLMT (Verbal Learning and Memory Test) depending on time (pre vs. post) and measure (subjective vs. objective) for **(A)** the “depressed” patient group (BDI-II ≥14) and **(B)** the “non-depressed” patient group (BDI-II <14). Bars represent standard error of mean. pre, preoperative assessment; post, postoperative assessment; VLMT, Verbal Learning, and Memory Test; FLEI, Fragebogen zur Geistigen Leistungsfähigkeit (Questionnaire for complaints of cognitive disturbances).

The repeated measures ANOVA for the pre- to postoperative comparisons of patients' memory performance (measure: objective vs. subjective) as well as their postoperative depressive symptoms (“depressed” vs. “non-depressed”) revealed significant main effects for both between-subject factors: measure [*F*_(1, 133)_ = 11.752, *p* = 0.001, *eta*^2^ = 0.081; objective memory > subjective memory] and depressive symptoms [*F*_(1, 133)_ = 20.887, *p* < 0.001, *eta*^2^ = 0.136; non-depressed > depressed]. Furthermore, an interaction effect between these two factors [*F*_(1, 133)_ = 14.695, *p* < 0.001, *eta*^2^ = 0.099] was found, showing significantly lower subjective memory scores than objective test results for only the “depressed” patient group but not for the “non-depressed” group. Figure 1 illustrates the pre- to postoperative change of subjective and objective memory scores as a function of depressive symptoms. Furthermore, a significant interaction effect between time and depressive symptoms was found [*F*_(1, 133)_ = 8.134, *p* = 0.005, *eta*^2^ = 0.058] indicating a significant decrease in depressive symptoms from pre- to postoperative assessment. The three-way interaction between time, depressive symptoms and measure was not significant [*F*_(1, 133)_ = 2.529, *p* = 0.114]. However, there was a tendency for the “depressed” patient group to indicate a negative change and underestimation of memory functions, while “non-depressed” patients tended to indicate a positive change in memory functioning whilst objective memory scores remained relatively stable.

### Memory, depressive symptoms, and seizure status

**Figure 2:**
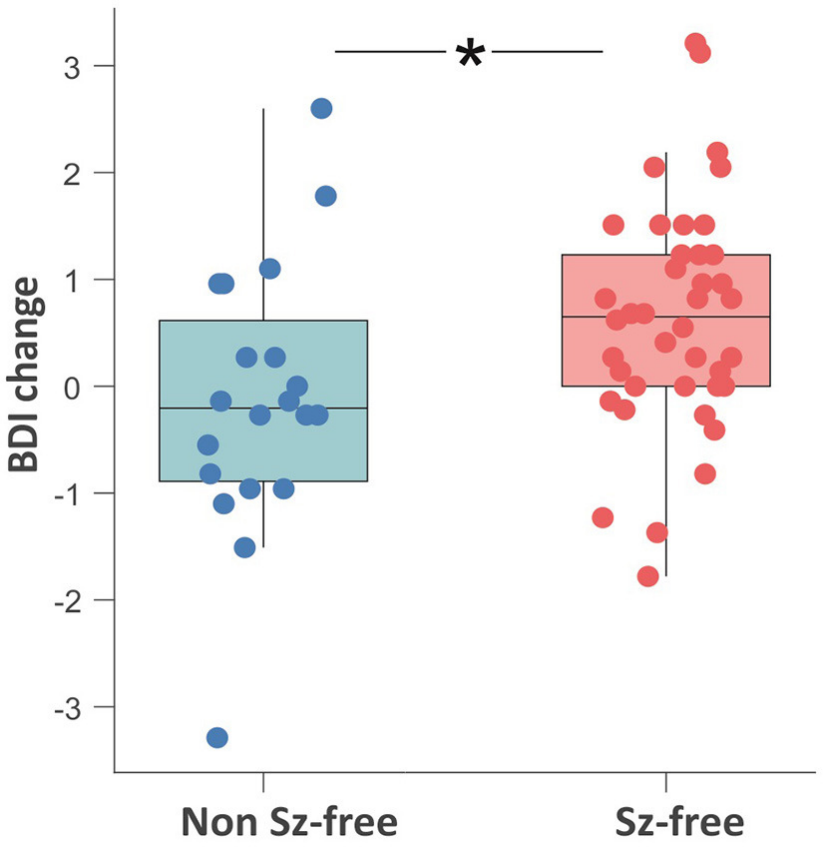
Boxplot showing the comparison of the pre- to postoperative change in depressive symptoms between the seizure-free (red) and non-seizure-free patients (blue). Change scores on the y-axis are computed as z-score_postoperative_ minus z-score_preoperative_. Negative values on the y-axis represent a postoperative increase of depressive symptoms. *p < 0.05; ns, non-significant; Sz, seizure; BDI, Beck Depression Inventory.

**Figure 3:**
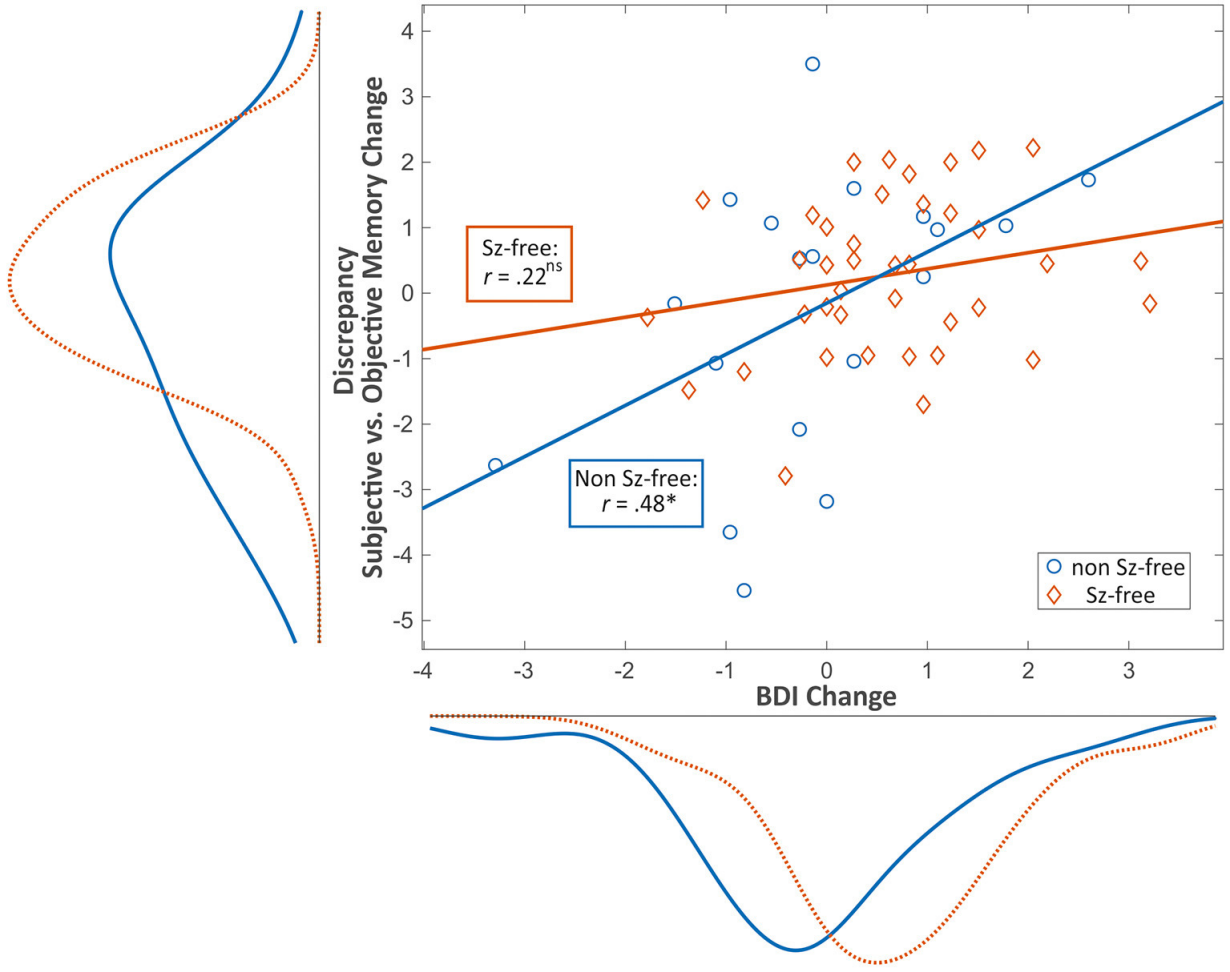
Scatter plot showing the relationship between pre- to postoperative change in depressive symptoms (x) and discrepancy (i.e., over-/ underestimation) between subjective and objective memory change (y) and the respective distribution curves for seizure-free (red) vs. non-seizure-free patients (blue). Values are given in standardized z-scores. Negative values on the y-axis represent overestimation whereas positive values represent underestimation of memory decline. Values near zero represent an adequate estimation of memory change. Positive values on the x-axis represent a postoperative decrease of depressive mood. *p < 0.05; ns, non-significant; Sz, seizure; BDI, Beck Depression Inventory.

Seizure-free patients reported higher BDI-II change scores (i.e., decrease of depressive symptoms) as compared to patients with persisting seizures [*t*_(58)_ = 2.43, *p* = 0.018; Figure 2]. Pearson product-moment correlation coefficients were computed to assess the relationship between the discrepancy of subjective vs. objective memory change on the one hand and change of depressive symptoms on the other hand. For the whole patient group, there was a moderate significant positive correlation between the two factors discrepancy and change in depressive symptoms (*r* = 0.369, *p* = 0.004) suggesting that a decrease in depressive symptoms was associated with a greater tendency to underestimate memory decline. Splitting the group based on seizure outcome, the positive correlation between the factors discrepancy and change in depressive symptoms was only found in the non-seizure-free group (*r* = 0.476, *p* = 0.040), while for the seizure-free patients, this correlation was not significant (*r* = 0.221, *p* = 0.176, Figure 3).

### Memory, depressive symptoms, and quality of life

**Figure 4:**
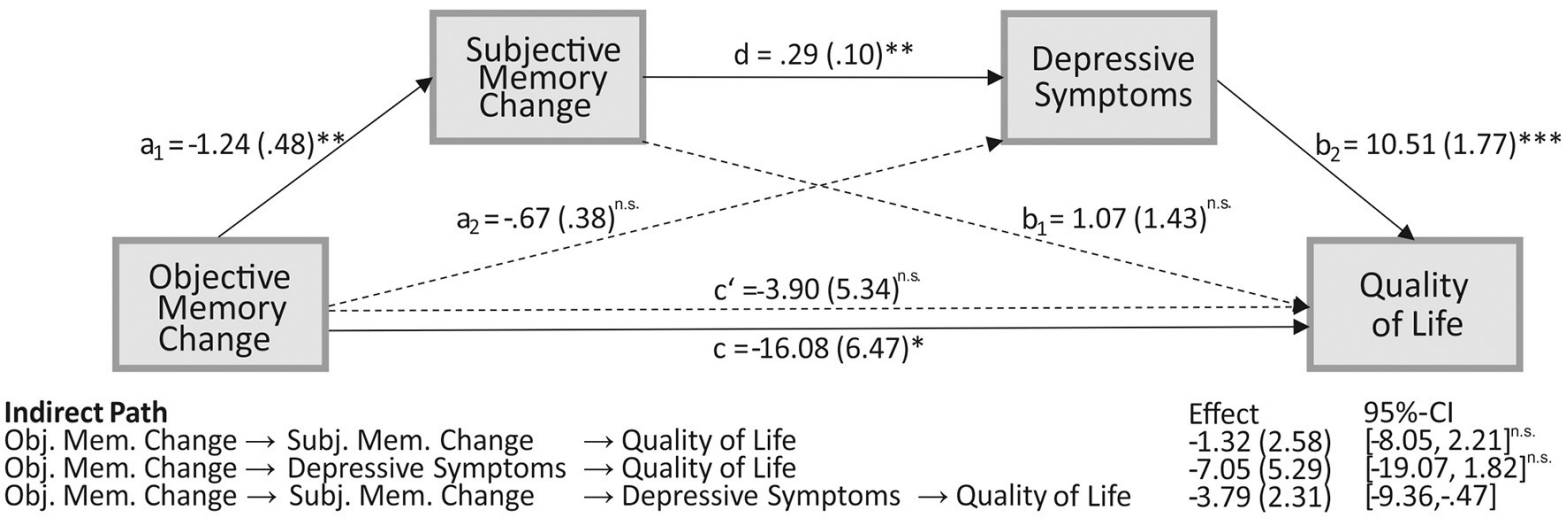
The serial mediating effect of subjective memory change and mood in the relationship between objective memory change and quality of life (QoL). All presented effects are unstandardized; a_n_ is the effect of objective memory change on mediators, objective memory decline is coded as 1 and no decline as 0; b_n_ is the effect of mediators on QoL; c is the total effect of objective memory change on QoL; c' is the direct effect of objective memory change on QoL after controlling for subjective memory change and depressive mood; d is the effect of subjective memory change on mood. *p < 0.05, **p < 0.01;***p < 0.001; ns, non-significant; obj., objective; subj., subjective; mem., memory.

Results from the serial mediation analysis indicated an indirect relationship between objective memory change and QoL through the two factors subjective memory change and depressive symptoms. In detail, as depicted in Figure 4, a deterioration in objective memory performance from pre- to postoperative assessment was associated with a subjective memory decline (a1 = −1.24, *p* = 0.012). This subjective memory decline was subsequently related to an increase in depressive symptoms (d = 0.29, *p* = 0.004), which on the other hand was related to a lower QoL (b2 = 10.51, *p* < 0.001). A 95% bias-corrected confidence interval based on 10,000 bootstrap samples indicated that this indirect effect through subjective memory change and depressive symptoms (a1 – d – b2 = −3.79) was entirely different from zero (95%- CI: [−9.36, −0.47]). In contrast, the indirect separate effects through both subjective memory change on QoL (a1 – b1), and depressive symptoms on QoL (a2 – b2), respectively, were not different from zero (−8.05 to 2.21 and −19.07 to 1.82, respectively; see Figure 4 for the associated effects). Since the total effect was significant (c = −16.08, *p* = 0.016) while the direct effect of objective memory change on QoL (c' = −3.90, *p* = 0.468) was non-significant, the relationship between verbal memory change and QoL is fully mediated through subjective memory change and depressive symptoms.

## Discussion

As a new approach, the present study investigated the mismatch between subjective and objective memory by taking into account the dynamic change in memory functioning as well as depressive symptoms after epilepsy surgery. Firstly, we found that patients with postoperative clinically relevant depressive symptoms were likely to underestimate memory performance. Secondly, for the postoperatively non-seizure-free patients, a postoperative decrease in depressive symptoms was associated with a tendency to underestimate memory decline. Thirdly, the relationship between objective memory change and QoL was mediated by the perception of memory change and depressive symptoms.

### Discrepancy between subjective and objective memory after epilepsy surgery

Despite the relevance of memory functioning in the context of epilepsy surgery, only a few studies investigated the change of subjective vs. objective memory functioning from pre- to postoperative time points. Critically, previous research has predominantly reported correlational evidence for an association between the judgment of memory functioning and emotional well-being or depressive symptoms (16, 17). For the first time, by only analyzing standardized values for both subjective and objective memory scores, we are able to report a quantitative measurement of this discrepancy that goes beyond correlational estimates. It shows a depression-related negative biased self-perception of memory functioning of roughly 1 to 1.5 standard deviations, which appears to be enormous considering the lack of such a bias in “non-depressed” patients. Our finding that clinically “depressed” patients underestimate their memory functions and tend to overestimate memory decline underlines the factor mood as crucial in the judgment of cognitive functioning. This is in line with our first hypothesis and other studies that identified psychopathological variables as crucial in explaining the discrepancy between objective test results and subjective judgments of memory functioning in PWE (4, 10–12). At the same time, this finding clearly indicates that patients' perceptions of memory functioning can be quite accurate in the absence of depressive symptoms. Taken together, our results, for the first time, offer a quantitative approximation of the previously reported mismatch between the perception and the objective performance of memory functioning from pre- to postsurgical assessment in PWE.

Since the nature of a neuropsychological examination in the epilepsy surgery setting (i.e., the assessment of patients at different time points before and after surgical intervention) allows for comparisons of change in specific symptoms or cognitive functions, we can appreciate the data in a dynamic way. By comparing standardized change scores, we found that patients with a decrease in postoperative depressive symptoms tend to underestimate their memory decline. Interestingly, this was only true for the non-seizure-free patients. Therefore, it seems that when patients are relieved of their burdens of having recurrent epileptic seizures, they may be less influenced by depressive symptoms when judging their memory changes in daily life. This matches previous findings demonstrating a subjectively reported relief from anxiety and worries in patients after epilepsy surgery (38, 39). Insofar, our results may be interpreted as a “honeymoon-” or “relief-effect” of seizure freedom and certainly add to the hypothesis that the positive effects of seizure freedom might even compensate for objective memory deficits (18). Interestingly, these effects are not only present at a specific pre- or postoperative timepoint but seem also to occur as a function of time in the two-year interval from pre- to postoperative assessment. In other words, patients – if seizure-free – do not seem to solely consider their current state when asked about their memory functions but are also able to perceive their change of memory functioning in a specific timeframe.

Furthermore, we found evidence for an association between a change in objective memory functioning and overall QoL after epilepsy surgery. This association was mediated by subjective memory change and depressive symptoms. More specifically, a decline in memory functioning was associated with a decreased QoL, but only when the perception of this decline led to an increase in depressive symptoms. When considering influencing factors of QoL after epilepsy surgery (20), the relationship between neuropsychological variables and QoL has mostly been described as nonexistent (40–42) or was only found in patients with continuing seizures (43). Analyzing different subjective factors in a comprehensive model allows for an explanation or specification of these earlier findings by highlighting the specific mechanisms underlying the association between objective memory change and QoL.

Our results, from a neuropsychological standpoint, suggest that for understanding the relevance of the impact of subjective memory change on the course of the disease and patients' QoL, as well as for the planning of treatment options, it is crucial to consider both, subjective memory functioning and depressive symptoms in the pre- and postsurgical evaluation of PWE. Since we found a strong concordance between subjective and objective memory functioning in the absence of depressive symptoms, the use of standardized memory questionnaires and norm scores (e.g., z-scores), in this case, may serve as a good estimate for memory functioning and, at the same time, allows for comparisons among different measures and time points. Furthermore, the availability of subjective data may help professionals to better recognize individual needs and worries with regard to changes after surgery, eventually leading to more patient-centered care and individualized presurgical counseling and risk assessment (44, 45). We, therefore, recommend the consideration of these variables both when interpreting neuropsychological results, for pre- and postoperative counseling and informing of patients, and for the individualized planning of psychological treatment options after surgery, e.g., guided by evidence-based intervention protocols, such as the HOBSCOTCH program (46). Finally, by considering the change in subjective memory functions and depressive symptoms, we have established a clear impact of objective memory performance on the QoL, which, at least from a patient's perspective, further underlines the clinical significance of self-rating instruments.

### Limitations and strengths

First, our patient sample was quite heterogeneous in terms of focus localization and lateralization. This may lead to decreased comparability of our results with results from other studies. On the other hand, we previously found that pertaining to frontal and temporal lobe epilepsies, patients with frontal lobe resections show a verbal memory decline that is, at least in some cases, comparable to memory loss after temporal lobe resections (26). This highlights the relevance of monitoring memory changes in patients with extra temporal lobe epilepsy, especially since this patient group becomes more important in the presurgical setting (22). Moreover, one might argue that subjective memory complaints may also pertain to autobiographical (explicit) memory deficits, or figural memory deficits while the VLMT and FLEI aim to assess verbal memory (generally processed by the left hemisphere). Therefore, it would be interesting to examine whether our results also hold true for memory functions processed more prominently by the right hemisphere. Interestingly, we found that when exploratively including the factor lateralization of epileptic focus in our repeated-measures ANOVA, the main effect of lateralization becomes significant (*p* = 0.026, *eta*^2^ = 0.038) while the interaction between lateralization and measure does not become significant (*p* = 0.267). We would like to stress, however that these effects should be interpreted with caution, since including this factor in our analysis led to small subgroups and small statistical power resulting in limited generalizability of these findings. Secondly, it has to be stressed that by assessing depressive symptoms with the BDI-II, we cannot infer a psychiatric comorbidity (29). However, this was not our primary aim; we were more interested in the quantitative degree of subjectively perceived depressive symptoms regardless of formal diagnostic boundaries. It would still be interesting to know whether PWE with high scores on the BDI-II also meet the criteria for a depressive mood disorder since this would have important treatment implications because of the clear effects on other health-related factors. Therefore, future studies should strive to investigate whether our results also apply to PWE that were diagnosed with a depressive mood disorder based on standardized assessments. Thirdly, because of the retrospective design of our study, QoL data was only available from postoperative assessment. Hence, we cannot be sure whether our patient sample experienced an overall improvement in QoL from pre- to postoperative assessment, as has been shown in other studies, at least for seizure-free patients (20, 47). It would have been interesting to compare the amount of QoL change in relation to subjective and objective memory change. Finally, one could argue that, since subjective memory, QoL, and depressive mood are related constructs, assessment of these factors by self-rating instruments (i.e., BDI-II, FLEI, QOLIE-31) would naturally yield high intercorrelations with the effect of causing the above-mentioned results. To address this issue, we did not analyze the merged scores of all subscales of these instruments, but we intended to measure only the most relevant and specific aspects of these constructs to prevent redundancy and shared variance caused by these intercorrelations (see Sections Objective Memory Measures, Subjective Cognitive Measures, Depressive Symptoms, Quality of Life).

We think that the appreciation of our data in a dynamic timeframe (i.e., *change* of symptoms in contrast to *presence/absence* of symptoms) is a strength of our study and represents a more realistic way of mirroring the course of symptoms or a disease. Our aim was to develop a comprehensive model of memory changes following epilepsy surgery beyond the incorporation of standard factors and analyses. By this means, and by computing standardized change and discrepancy scores, we were able to present data in a flexible and appealing manner to better understand the impact of epilepsy surgery on a subjective level. For example, with this approach, we could quantify the discrepancy between objective and subjective memory, which goes beyond reporting standard correlational data. Future studies should adapt this approach and include other factors of cognitive functioning as well. In addition, future studies should compare patients' progress of (subjective) memory and QoL change with a control group and establish criteria for clinically meaningful change.

## Conclusion

Due to changes in epilepsy-related factors, such as fluctuations in seizure frequency, or cognitive changes after epilepsy surgery, epilepsy as a condition is characterized by dynamic changes in symptomatology in the course of the disease. Therefore, it is necessary to study and comprehend these alterations accordingly. By implementing a dynamic approach, we demonstrated a pronounced depression-related discrepancy between subjective and objective memory by showing a negative biased self-perception of memory change after epilepsy surgery. Considering the lack of such a bias in “non-clinically depressed” patients, it reflects the factor mood as crucial in the evaluation of pre- and postoperative cognitive functioning in PWE. Moreover, postoperatively “non-clinically depressed” patients tended to underestimate their memory decline, but only when they were seizure-free after surgery, possibly indicating a relief-effect of seizure freedom. Finally, we found evidence for an association between objective memory change and overall QoL after epilepsy surgery, which was mediated by subjective memory change and depressive symptoms, highlighting mood and memory functioning as crucial factors in the pre- and postsurgical evaluation of PWE. Based on these findings, we plead for the broader consideration of standardized self-rating instruments, even in longitudinal observations of non-surgical patients, with the goal of better interpreting crucial changes in the course of the disease and how these changes may affect patients' QoL.

## Data availability statement

The raw data supporting the conclusions of this article will be made available by the authors, without undue reservation.

## Ethics statement

The studies involving human participants were reviewed and approved by the Local Ethics Committee of University of Bielefeld, Germany, no. 2016-001. The patients/participants provided their written informed consent to participate in this study.

## Author contributions

FM and PG contributed to conception and design of the study, organized the database, performed the statistical analyses, and generated/ prepared the figures. FM wrote the first draft of the manuscript. PG wrote sections of the manuscript. FM, PG, CB, and MH contributed to manuscript revision, read, and approved the submitted version. All authors contributed to the article and approved the submitted version.

## Funding

PG holds a Junior-Professorship at the Bielefeld University endowed by the von Bodelschwinghsche Stiftungen Bethel. The funding source did not have any influence on the study's design, data collection, analyses, interpretation, manuscript preparation, and submission.

## Conflict of interest

The authors declare that the research was conducted in the absence of any commercial or financial relationships that could be construed as a potential conflict of interest.

## Publisher's note

All claims expressed in this article are solely those of the authors and do not necessarily represent those of their affiliated organizations, or those of the publisher, the editors and the reviewers. Any product that may be evaluated in this article, or claim that may be made by its manufacturer, is not guaranteed or endorsed by the publisher.
